# Pharmacy Practice and Education in Estonia

**DOI:** 10.3390/pharmacy7030087

**Published:** 2019-07-10

**Authors:** Daisy Volmer, Kristiina Sepp, An Raal, Jeffrey Atkinson

**Affiliations:** 1 Institute of Pharmacy, Faculty of Medicine, University of Tartu, Nooruse 1, 50411 Tartu, Estonia; 2 Pharmacolor Consultants Nancy, 12 rue de Versigny, 54600 Villers, France

**Keywords:** pharmacy, education, practice, Estonia, European Union

## Abstract

The Pharmacy Education in Europe (PHARMINE) project studied pharmacy practice and education in the European Union (EU) member states. The work was carried out using an electronic survey forwarded to selected pharmacy representatives at community and hospital pharmacies, in the pharmacy industry and at drug authorities. The surveys of the individual member states are now being published as reference documents for students and staff interested in research on pharmacy education in the EU, and in mobility. This paper presents the results of the PHARMINE project on pharmacy practice and education in Estonia. In this paper, we examine the harmonisation of practice and education in Estonia with EU norms. Community pharmacies in Estonia provide traditional and extended services, of which influenza vaccination, the evaluation of the risk of diabetes, and medication use review have been introduced recently. Pharmacists (in Estonian *proviisor*) study at the University of Tartu for five years and graduate with a Master of Pharmacy (MSc Pharm) degree. A pharmacist can be the owner of a pharmacy, or work as a pharmacy manager or chief pharmacist in either a community or a hospital pharmacy. Assistant pharmacists (in Estonian *farmatseut*) study at the Tallinn Health Care College for 3 years; after graduation, they are mainly employed in community pharmacies. The University of Tartu is the only university in Estonia providing higher education in pharmacy at university level. The pharmacy curriculum is an integrated (bachelor followed by master), pharmaceutical product-oriented study programme. It was last updated in 2019. On that occasion, several changes were made such as the introduction of competency-based modules; novel methods in education and training based on the constructive alignment and the restructuring of the six-month traineeship. Several new courses focus on the concepts of clinical pharmacy and on patient-centred communication. In the current pharmacy curriculum, there is a balance between chemical and medical subjects. The traineeship is provided for six months at a community and/or hospital pharmacy in the 5th year. Currently, the pharmacy curriculum at the University of Tartu does not offer specialization in subjects such as hospital or industrial pharmacy.

## 1. Introduction

The Pharmacy Education in Europe (PHARMINE) project surveyed the state of pharmacy practice and education in the member states of the European Union (EU), including Estonia, between 2008 and 2011, with an update in 2017–2019. The methodology used in the PHARMINE project and the main results obtained have already been published [1]. In the first part of the study, the PHARMINE project gathered information on community pharmacy practice, and on specialised hospital and industrial pharmacy practice, as well as the education and training necessary. The PHARMINE project also dealt with other personnel working in pharmacies such as assistant pharmacists: their education, training and responsibilities.

The PHARMINE project went on to study the legal and administrative context of pharmacy practice and education. In the EU, pharmacy practice and education fall under two jurisdictions: European and national. EU legislation is confederal in structure. The freedoms of movement and of exercise of the profession are the cornerstones. To ensure this, there is a system of automatic recognition of professional qualifications for sectoral professions such as pharmacists. For regulated professions in specific sectors—doctor, dentist, pharmacist, general care nurse, midwife, veterinary surgeon, architect and lawyer—recognition of the qualifications of a professional coming from a given EU member state in another EU member state is regulated by EU sectoral directives (for pharmacists, etc.) that are specific to each profession.

To work in another EU member state, professionals need to apply to the relevant authority of that country, providing proof of the qualifications obtained in their home member state. Such procedures are regulated by directives issued by the European Commission of the EU. The latter are ordinances laying down the broad imperatives on the practice and education of the given profession [2]. An EU directive requires member states to achieve a particular result—in this case, the harmonisation of pharmacy practice and education—without dictating the means of achieving that result. Thus, directives leave the different member states with leeway as to the exact rules to be adopted. The result of this is that member states have systems that are more or less harmonised with the EU directive paradigm.

In parallel to the above EU pan-national system, member states may introduce national legislation relating, for example, to specialised practice, and to the ownership and management of pharmacies.

Pharmacy education and training in Europe is also influenced by the Bologna agreement on the harmonisation of European degree courses, and student and staff exchange [3]. The Bologna agreement was signed by the education ministers of the governments of the European Higher Education Area (48 members including the 28 EU member states). It proposes recommendations that are not legally binding. The first of these is a harmonized structure for all university degrees (including pharmacy) with a bachelor (3 years) followed by a master (2 years) degree. In this aspect, the Bologna agreement is in opposition to the EU directive. The latter requires a five-year, “tunnel” degree structure for pharmacy, i.e., a degree course that offers no possibility for intermediate entry or exit after accomplishment of a three-year bachelor period.

The idea behind the Bologna recommendations is to improve student mobility, with the development of tools to promote student exchange programmes like the European Credit Transfer and Accumulation System (ECTS). This provides credits to students for defined learning outcomes. ECTS are listed in a Diploma Supplement that describes the nature, level, context, content and status of the studies that were successfully completed by a student. This system allows students to validate at their home university, studies carried out at their host university.

This paper looks at how the EU directive and the Bologna recommendations apply in Estonia, a post-soviet country which regained its independence in 1991. Estonia has been a member of the EU since 2004 [4].

In order to place pharmacy practice within the general health situation in Estonia compared to Europe, it is important to highlight that the health care system and its development are influenced by the changes within society. In Estonia and other western countries, the population is aging, decreasing in numbers, and increasing in mobility. As a result of increased health awareness, in diagnostic options, and an improvement in the quality of life, expectations of the population are increasing.

The Estonian population was 1,323,824 as of 1 January 2019 [5]. The average life expectancy in Estonia has increased in recent years to 82 for women and 73 for men; this increase in average life expectancy has occurred as a result of lower mortality, due to more effective treatment methods. Albeit, compared to the EU average, life expectancy in Estonia is shorter—by 5.5 years for women and 9 years for men. In 2016, Estonia was the penultimate of the 28 EU member states in terms of healthy life years for men; for women, Estonia came in 18th [5,6]. Expenditure on health has been stable but low compared to the EU average: 6.5% versus 9.4% of gross domestic product (GDP) [7]. In 2017, specialized medical care costs were €629.1 million and basic medical care costs were €113.6 million. The cost of medicines was €126.6 million and medical supplies was €9.5 million. Public funding covered 98% of health care costs in 2017 [8]. In order to improve the situation of public health, the engagement of all public and private stakeholders—including pharmacists—is required.

## 2. Design

Information was obtained from academics and practicing pharmacists (the first 3 authors of this article) and from internet sources on:

- pharmacy:
  ○ practice (community, hospital and industrial);
  ○ legislation;
  ○ education and training.
- harmonisation with the EU sectoral directive on pharmacy [2] and with the Bologna recommendations [3].

Electronic survey methodology was used; data was collected in 2010 and revised in 2017 and 2019. Sampling was performed by sending the survey to the members of the Tartu University Institute of Pharmacy, associations of industrial, community, hospital, and other specialized pharmacists, and the Estonian association of pharmacy students. Collection of data took between six and 12 months. Data collection was performed electronically using standard survey platforms. We attempted at all times to collect objective (if possible numerical) data.

The information is presented in the form of tables in order to facilitate legibility. This type of presentation was developed in association with the journal’s editorial board and has been described in detail in a previous publication [9]. This format will ease the comparison of different EU countries by students and staff envisaging exchange programmes, and by researchers in pharmacy education and practice.

## 3. Evaluation and Assessment

### 3.1. Organisation of the Activities of Pharmacists, Professional Bodies

Table 1 provides details of the numbers and activities of community pharmacists and pharmacies in Estonia. Items are expounded in the “comments” column, which also includes opinions and trends for the future.

Using the data in reference [1] and Table 1, it can be calculated that compared to the EU linear regression estimation (for definition and calculation see reference [1]), the ratio of the number of community pharmacists in Estonia to the population compared to the EU linear regression estimation = 1.03. Thus, the number of pharmacists per population is similar to the EU norm. The same comparison for community pharmacies produces a ratio of 1.28, higher than the EU norm. The number of pharmacists/pharmacies of 1.8 is slightly lower than the EU average of 2.1 ± 0.7 [1].

The activities and occupations of pharmacists in Estonia are similar to those of community pharmacists in other EU member states [1]. In recent years, community pharmacies in Estonia have started to provide more extended services such as health screening tests—monitoring of blood pressure, blood sugar and cholesterol [10]. The first influenza vaccination campaign was launched in October 2018 and, in one month, approximately 10,000 people were immunized at community pharmacies [11]. A pilot project titled “Medication use review at community pharmacies” was initiated in February 2019. This is a pilot project aimed at clarifying the professional competency of pharmacists and assessing the impact of this service on the awareness of medication among patients.

One of the traditional and core competencies of pharmacists—the compounding of medicines—has decreased: in 2017, only 4% of community pharmacies prepared extemporaneous medicines [12]. The pharmacy has moved from a product-oriented to a patient-oriented practice with a new focus on patient care and quality provision of both traditional and extended community pharmacy services.

Table 2 provides details of the numbers and activities of specialists other than pharmacists working in community pharmacies in Estonia.

Table 3 provides details of the numbers and activities of hospital pharmacists and pharmacies in Estonia.

Table 4 provides details of the numbers and activities of pharmaceutical industries and pharmacists in industry, and in the other sectors, in Estonia.

Table 5 provides information on professional associations for pharmacists in Estonia in terms of territorial restrictions, ethical principles and the role of pharmacists.

### 3.2. Pharmacy Faculties, Students, and Courses

At the University of Tartu, an independent Institute of Pharmacy was opened in 1842 and, since 1919, a Pharmacy programme has been provided in Estonian. The Soviet era was characterized by the politicization of teaching and research: a number of ideological subjects and military instruction were included in the Pharmacy curriculum. Difficulties were encountered in gaining access to foreign literature, and research became increasingly an activity within the Soviet Union. In the 1960s, Tartu was an important pharmacy centre in the Soviet Union. The restoration of Estonia’s independence in 1991 brought a number of changes to the Pharmacy curriculum. The latter has been frequently updated—the last time, in 2019. In comparison with previous curricula, the current programme includes more medicinal subjects and interactive study methods. The Pharmacy Institute has participated in research funded by the European Union in the field of pharmacy and quality assurance (PHARMINE and PHAR-QA). The Pharmacy curriculum has been internationally accredited in 2001, 2007 and 2014.

Table 6 provides details of pharmacy higher education institutions (HEIs), staff and students in Estonia.

A comparison to the EU average for staff shows that Estonia has a low ratio 0.40 [1], although the number of pharmacy HEIs is higher than the EU norm at 2.08.

Table 7 provides details of past and present changes in pharmacy education and training in Estonia.

### 3.3. Teaching and Learning Methods—Student Hours

Table 8 represents teaching and learning methods in student hour.

The number of lectures and practicals are almost equal in years 1–3. The number of practicals decreases and number of tutorials increases in the 4th year. The research project and traineeship are placed in the 5th year.

### 3.4. Subject Areas

Table 9 represents subject areas in student hours. For definitions of the subject areas, see Reference [1].

Years 1 and 2 are devoted mainly to providing basic knowledge in chemical and medical sciences. Years 3 and 4 involve in addition pharmaceutical technology. Social sciences are taught during the entire pharmacy curriculum. The research project and six-month traineeship at a community and/or a hospital pharmacy are placed in year 5. According to the new pharmacy curriculum, students can take traineeship as elective subjects in other pharmacy sectors, e.g., pharmaceutical industry, wholesale companies, governmental institutions dealing with medicines during years 2–4.

The overall ratio of MEDISCI/CHEMSCI (years 1–5) is 1.3. It is similar to that of other HEIs in the EU which have a “balanced” course i.e., a medicinal sciences/chemical sciences index of 1.2. Other member states have more “medical” courses, such as Ireland and the Netherlands, with indices of 2.6 and 1.6, respectively [1].

### 3.5. Impact of the Bologna Principles

Table 10 provides details on the impact of the Bologna agreement [3] on the Institute of Pharmacy, University of Tartu, Estonia.

Estonia like other EU countries has adopted some elements of the Bologna agreement such as the use of ECTS and comparable degrees with a diploma supplement. During the updating of the pharmacy curriculum in 2018, to maintain the five-year tunnel degree was decided. However, discussions on how to provide master’s degree education at the University of Tartu for assistant pharmacists graduating from the Tallinn Health Care College will start in the near future.

### 3.6. Impact of European Union (EU) Directive 2013/55/EC

Table 11 provides details on the various ways in which the EC directive [2] impacts on pharmacy education and training in Estonia.

Pharmacy education in Estonia conforms to the different aspects of the EU directive with a five-year degree and a six-month traineeship.

## 4. Discussion and Conclusions

Community pharmacies in Estonia, providing traditional and extended services, could be more integrated into the health care system. In 2019, it is expected that the Estonian government will approve the ten-year “Public Health Development Plan”, which will suggest new directions and recommendations for actions at different levels of quality health care. This development plan is based on the government’s priority to improve life expectancy and the quality of life [24]. The changes envisaged will have an effect on pharmacy practice and more general on the pharmacy profession. What the public expects from pharmacists goes beyond the dispensing and compounding of medicines. For instance, patients with chronic conditions or polypharmacy are looking for health counselling, self-care consultation and management of medicines. The improvement in vaccination coverage and the tackling of resistance to vaccines have already shown that pharmacists have an added value in the eyes of the public [10,11,12].

Unfortunately, there has been little governmental involvement in the development of pharmacy practice. Thus, professional organizations have taken the lead in development of the quality standards for community and hospital pharmacies and of new innovative solutions in different pharmacy settings [25].

The University of Tartu is the only university in Estonia providing higher education in pharmacy. The pharmacy curriculum is a pharmaceutical product-oriented study program. After several changes (in 1997, 2003, 2007, 2019) different new courses have been introduced focusing on clinical pharmacy and patient-centred communication. In the current pharmacy curriculum, there is a balance of chemical and medical subjects [26]. The traineeship is provided for six months during the 5th year of study mostly at community pharmacies but also in hospital pharmacies. Currently the pharmacy curriculum at the University of Tartu does not offer specialization.

In 2017, the pharmacy curriculum was evaluated using the PHAR-QA framework for competency-based education [26] with feedback from teaching staff and students at the University of Tartu as well as from different pharmacy stakeholders such as community pharmacists, hospital pharmacists, industrial pharmacists and drug authority experts. Representatives of different pharmacy sectors addressed several issues in the pharmacy programme organisation and training methods and suggested several solutions:

- increased collaboration between different health care professionals,
- more integrated training and common courses with medical students,
- more patient care competencies linking the pharmacy programme with practice,
- more reflection and practical implementation of theoretical knowledge,
- broader use of problem-based learning,
- introduction of business and entrepreneurship subjects to the pharmacy programme, and
- enhanced requirements for pharmacy students and for internship supervisors at community and hospital pharmacies given that pharmacy internship plays a very important role in implementing of professional competencies.

Representatives of academia and other pharmacy stakeholders concluded that although the existing pharmacy programme is designed to provide broad theoretical knowledge, more efficient training methods should be implemented and specialists from different fields of pharmacy practice should participate in the teaching process, thus more efficiently linking theory with practice [26]. The issues described above have been addressed when updating the pharmacy curriculum in 2019.

The main conclusion of this survey is that pharmacy education and training in Estonia follows EU directives and principles of Bologna agreement. Based on the PHAR-QA framework for competency-based education, the pharmacy curriculum was updated in 2019 and corresponds to the “Pharmacist´s occupational qualification standards”. In collaboration with pharmacy stakeholders, we will soon start to harmonise pharmacy education in Estonia. Furthermore, the implementation of new education and training principles will be closely integrated with collaborative communication and future changes in the pharmacy sector.

## Figures and Tables

**Table 1:** Numbers and activities of Estonian community pharmacists and pharmacies [12,13,14,15].

Source	Number/Reply	Comment
Community pharmacists	894 in 2019	A total of 41% of the community pharmacy staff are pharmacists. The total number of employees is 2180.
Community pharmacies	495 in 2019	A total of 343 are general and 152 are branch pharmacies. The main difference with branch pharmacies is the absence of any obligation to compound medicines. A branch pharmacy operates under the supervision of a general pharmacy. Starting in April 2020, branch pharmacies operating in communities with a population of >4000 will be either transformed into general pharmacies or closed.
Competencies and roles of community pharmacists		A pharmacist can be the owner, manager and responsible pharmacist. The main professional tasks are related to organization and provision of high-quality pharmaceutical care. Professional competencies include: dispensing and counselling of prescription and over-the-counter (OTC) medicines, compounding/preparation of extemporaneous medicines, point of care testing (e.g., blood pressure measurement, cholesterol, blood sugar, haemoglobin), disease prevention and health education of the patients, reporting of adverse drug reactions (pharmacists are not authorized to report, but assist patients in reporting of ADRs), and piloting extended services (e.g., medication use review, influenza vaccination, screening for the risks of diabetes, advice on cessation of smoking).
Is ownership of a community pharmacy limited to pharmacists?	Yes	Starting on 1 April 2020, >50 per cent of the shares of the private legal entity (the dominant influence) must be in the possession of a pharmacist, working as the manager in at least one of the general pharmacies (up to a maximum of four general pharmacies).
Rules of geographical distribution of community pharmacies	Yes	Pharmacies in a community with a population of <4000 can operate as branch pharmacies.
Are drugs and other health care products available to the public through other channels?	Yes/No	Community pharmacies have a monopoly on the sale of prescription and OTC medicines. Health care products are available in supermarkets, etc. In 2013, an online pharmacy, operating as a branch pharmacy, was created.

**Table 2:** Numbers and activities of other personnel working in community pharmacies in Estonia [12,13,14,15].

Source	Number/Reply	Comment
Are other persons involved in community pharmacy practice?	Yes	Assistant pharmacists and customer service specialists.
Their titles and numbers	774 in 2019	Assistant pharmacists form 33% of the community pharmacy workforce. Other personnel such as customer service specialists and cleaning workers form 26% of the community pharmacy workforce. People working in such positions do not have any special training. A customer service specialist is responsible for counselling of other goods (not medicines). It is common for pharmacy students to work as a customer service specialist.
Duration of study for assistant pharmacists	3 years	Professional higher education, Tallinn Health Care College.
Subject areas		Basics of chemistry, pharmaceutical technology, pharmacology and social pharmacy, practical training on dispensing and counselling of medicines. This curriculum is highly practice oriented.
Competencies and roles		The main professional tasks are related to the organization and provision of high-quality pharmaceutical care. Professional competencies include: dispensing of and counselling on OTC medicines, compounding/preparation of extemporaneous medicines, point of care testing (e.g., blood pressure measurement, cholesterol, blood sugar, haemoglobin), and disease prevention and health education, As opposed to pharmacists, assistant pharmacists cannot hold the position of pharmacy manager.

**Table 3:** Numbers and activities of hospital pharmacies and pharmacists in Estonia [12,13,14,15,16].

Source	Number/Reply	Comment
Hospital pharmacists	71 in 2018	At the end of 2018, 71 pharmacists, 39 assistant pharmacists and 39 other personnel worked in hospital pharmacies. On average, a hospital pharmacy has five employees.
Hospital pharmacies	24 in 2019	
Competencies and roles of hospital pharmacists		Professional competencies: part of a multidisciplinary patient-care team, redaction of a review of the use of medicines, supply and compounding of medicines, production of patient-specific medicines (e.g., cytotoxic preparations), and management of the drug formulary.

**Table 4:** Numbers and activities of pharmaceutical industry and pharmacists in industry and in other sectors in Estonia [14,17,18].

Source	Number	Comment
Companies with some aspects of production, R&D and distribution	21 (2019)	Manufacturers of human and veterinary medicinal products—8, Manufacturers of active substances—1, Repackaging and labelling—12 companies.
Companies with distribution only	64 (2019)	A total of 76% of the market volume of human medicines is provided by three major wholesalers: Magnum Medical OÜ (28%), Tamro Eesti OÜ (27%), and Apteekide Koostöö Hulgimüük (21%). Compared to the previous year, the market volume of pharmaceuticals increased by 8.1% in 2018, amounting to € 325 million. Most wholesalers delivered medicines to community pharmacies—€ 228.0 million (70% of market volume). Sales to hospital pharmacies accounted for 29% (€ 93.6 million) and sales to other institutions 1%, of market volume (€ 3.8 million). The turnover of prescription medicines was 85% of the volume of the pharmaceutical market [16]. Out of the 64 companies, five are wholesalers of veterinary medicines.
Pharmacists working in industry	*Circa* 120	Official statistics not available.
Competencies and roles of pharmacists working in industry		Professional competencies: marketing, distribution, drug evaluation, and marketing authorization and management.
**Other sectors**		
Pharmacists working in other sectors	*Circa* 200	Official statistics not available.
Sectors which pharmacists are employed		Governmental institutions: ○ State Agency of Medicines, ○ Ministry of Social Affairs, ○ Health Board, ○ National Institute for Health Development, and ○ Health Insurance Fund. HEIs: ○ University of Tartu, ○ Tallinn Health Care College, and ○ Tartu Health Care College. Other: ○ companies providing services for drug marketing, ○ drug marketing authorization and monitoring of clinical studies, ○ start-up companies related to medicines, and ○ armed forces.
Competencies and roles of pharmacists employed in other sectors		Armed forces—supply of medicines, Universities, schools of professional higher education ○ education of pharmacists and assistant pharmacists, and ○ conducting pharmacy research. national health services, governmental institutions dealing with medicines ○ development and surveillance of pharmacy legislation.

**Table 5:** Professional associations for pharmacists in Estonia [14,15,19,20].

Source	Reply	Comment
Registration of pharmacists	Yes	Pharmacists and assistant pharmacists providing pharmacy services in Estonia must be recorded in the national register of pharmacists and assistant pharmacists, kept by the Health Board. A license holder (community or hospital pharmacy) is responsible for informing the Health Board of the employment or dismissal a pharmacist or assistant pharmacist.
Creation of community pharmacies and control of territorial distribution	Yes	As from 1 April 2020, a community pharmacy can be run only by a private legal person (pharmacist) holding, > 50 per cent of the shares of the private legal entity. A pharmacy license holder can manage up to four general pharmacies operating in a community with a population of >4000. Shareholders or members of a private legal entity holding a general pharmacy authorization must not include persons holding a wholesale distribution or manufacturing authorization, or a health service authorization. The State Agency of Medicines supervises the holders of a pharmacy license.
Ethical considerations and role of pharmacists	Yes	In 2012, a council composed of representatives of professional pharmacy organizations, of the State Agency of Medicines, and of the University of Tartu proposed quality guidelines for community pharmacy services; these were updated in 2016. In November 2016, the occupational qualification standards for pharmacists and assistant pharmacists were approved. As an extension, an updated version of the Code of Professional Ethics for Pharmacists was included with the standards. Overall the guidelines aim to formulate the principles of modern pharmacy services, i.e., the aspects of quality pharmacy services, and to define clear criteria for evaluating the quality of pharmacy services. The guidelines give all pharmacists the opportunity to evaluate the working of their pharmacy and improve the efficiency of their services.
Quality assurance and validation of HEI courses for pharmacists	Yes	The curriculum at the University of Tartu is subjected to international accreditation every seven years.
Professional continuing education courses for pharmacists	Yes	Starting in January 2015, it is compulsory for practicing pharmacists to take continuous professional development (CPD) courses at a rhythm of at least 40 academic hours every two years. Professional training can be participation in CPD courses, seminars, conferences, etc.

**Table 6:** Pharmacy higher education institutions (HEIs), staff, and students in Estonia [21,22,23].

Source	Number/Reply	Comment
Total number of HEIs for pharmacy	2	In Estonia there is only one pharmacy school providing higher education in pharmacy on university level—the University of Tartu. In addition, Tallinn Health Care College provides professional higher education (3 years) for assistant pharmacists. The following sections describe the information concerning the Institute of Pharmacy at the University of Tartu only.
**Tartu-Estonia** Public HEIs	1	
Attached to a medical faculty	Yes	
Do HEIs offer B.Sc. and M.Sc. degrees?	Yes	The integrated B+M curriculum for pharmacy provides a tunnel degree (5 years) for continuous education. No specialization is available during pharmacy training.
Do HEIs offer a M.Sc. Pharm. Degree after a B. Sc. degree in another discipline?	Yes	There is a distance learning system (3 years of study) for assistant pharmacists graduating from the Tallinn Health Care College who wish to become pharmacists.
**Teaching staff**		
Number of teaching staff (nationals)	18	Includes seven full-time and 11 part-time staff with one visiting professor from the US (social pharmacy).
Number of professionals from outside the HEIs, involved in education and teaching	*Circa* 35	Professionals with a medical background are involved in teaching of courses such as bioethics, pharmaco-epidemiology and pharmaco-economics. Pharmacists employed at the State Agency of Medicines, community and hospital pharmacists intervene as visiting lecturers (*n* = 10). Pharmacists intervene as supervisors of traineeships at community and hospital pharmacies (circa 20–25).
**Students**		
Number of places at entry following secondary school	36	
Applicants per place	*Circa* 150	Approximately 3.5–4 applicants for each place.
Number of graduates that become professional pharmacists	*Circa* 20–25	
**Entry requirements following secondary school**		
Specific pharmacy-related, national entrance examination	No	Based on the results of secondary school examinations: mathematics, chemistry or biology (70%) and mother tongue (Estonian) (30%).
Is there a national *numerus clausus*?	No	
**Fees**		
Home students	0 €	
Distance learning	*Circa* 2500€ per year	

**Table 7:** Past and present changes in education and training in Estonian pharmacy HEIs.

Item	Reply	Comments
Major changes before 2019 in Estonia	Yes	Establishment of the Council of the Institute of Pharmacy following structural reforms at the Faculty of Medicine at the University of Tartu (2015) Competency based evaluation of the pharmacy curriculum (2016–2017) Restructuring and updating of pharmacy traineeship, including use of e-portfolio to support independent learning by students (2017) Development of competency-based modules for the pharmacy curriculum (2018–2019) Updating of the pharmacy curriculum including novel teaching and assessment methods (2019)

**Table 8:** Teaching and learning methods in student hours *.

	Year 1	Year 2	Year 3	Year 4	Year 5	Total
Lecture	220	210	230	230	60	950
Tutorial	175	110	140	300	86	811
Practical	275	310	300	150	-	1035
Project work	-	-	-	-	182	182
Traineeship Community	-	-	-	-	480	480
Subtotal	670	630	670	680	808	3458
Electives	50	50	50	50	20	220
Optional	30	30	30	30	-	120
Total	750	710	750	760	828	3798

* Calculation of student hours: 1 ECTS (European Credit Transfer System) = 26 h (13 h formal teaching + 13 h independent work) for obligatory subjects; 1 ECTS = 20 h (10 h formal teaching + 10 h independent work) for electives. In Table 8 only formal teaching hours are presented.

**Table 9:** Subject areas in student hours *.

	Year 1	Year 2	Year 3	Year 4	Year 5	Total	%
CHEMSCI	221	325	195	39	-	780	22
PHYSMATH	-	-	26	-	-	26	1
PHARMTECH	-	65	286	169	-	520	15
MEDSCI	300	260	340	120	72	1013	29
LAWSOC	117	78	-	143	-	338	10
GENERIC + traineeship	14	25	-	14	728	781	23
Total						3458	100

* Calculation of student hours: 1 ECTS = 26 h (13 h formal teaching + 13 h independent work) for obligatory subjects. In Table 9, only formal teaching hours are presented. CHEMSCI: chemical sciences; PHYSMATH: physical and mathematical sciences; PHARMTECH: pharmaceutical technology; MEDISCI: medicinal sciences; LAWSOC: law and social sciences; GENERIC: generic competences and traineeship.

**Table 10:** Ways in which the Bologna agreement impacts the Estonian pharmacy HEI.

Item	Reply	Comments
“Comparable degrees with diploma supplement”	Yes	The Diploma Supplement is issued systematically in Estonian, and if needed in English.
“Two main cycles (B and M) with entry and exit at B level”	No	At the University of Tartu, the pharmacy studies are not divided into Bachelor and Master cycles. However, professional higher education provided at Tallinn Health CareCollege is in principle comparable with a “Bachelor level” of pharmacy studies at university.
“European Credit Transfer System (ECTS) system of credits”	Yes	The ECTS system was adopted in autumn 2009. However, the ECTS system is not linked to lifelong learning system.
“Addressing obstacles to mobility”	Yes	The rigid chronology of the course system complicates mobility. To solve the problem, a mobility window (up to 3 months of study abroad) is planned. Language skills and funding are other barriers.

**Table 11:** Ways in which elements of the European Commission (EC) directive impacts on pharmacy education and training in Estonia.

The Directive States	How Does/Will This Directive Statement Affect Pharmacy Education and Training?
“Evidence of formal qualifications as a pharmacist shall attest to training of at least five years’ duration ...” “ .. four years of full-time theoretical and practical training at a university or at a higher institute of a level recognised as equivalent, or under the supervision of a university;” “…six-month traineeship in a pharmacy which is open to the public or in a hospital, under the supervision of that hospital’s pharmaceutical department.” “The balance between theoretical and practical training shall, in respect of each subject, give sufficient importance to theory to maintain the university character of the training”	The pharmacy curriculum is 5 years and this requirement is fulfilled. Our students have 4 years theoretical and practical training at the university and this requirement is fulfilled. Our students perform the six-month traineeship. At least three months in a community pharmacy and the remaining three months at a community or hospital pharmacy. From the point of view of the university, it is important to focus on theoretical knowledge in order to prepare the students for further studies (Ph.D.). The skilful combination of theory linked to practice is a very important tool in a quality teaching process.
